# Chronobiology of Parkinson's disease: Past, present and future

**DOI:** 10.1111/ejn.15859

**Published:** 2022-11-23

**Authors:** Ziba Asadpoordezaki, Andrew N. Coogan, Beverley M. Henley

**Affiliations:** ^1^ Department of Psychology Maynooth University Maynooth Co Kildare Ireland; ^2^ Kathleen Lonsdale Institute for Human Health Research Maynooth University Maynooth Co Kildare Ireland

**Keywords:** chronotherapy, circadian clocks, circadian rhythm, neurodegenerative diseases, Parkinson's disease, sleep–wake disorders

## Abstract

Parkinson's disease is a neurodegenerative disorder predominately affecting midbrain dopaminergic neurons that results in a broad range of motor and non‐motor symptoms. Sleep complaints are among the most common non‐motor symptoms, even in the prodromal period. Sleep alterations in Parkinson's disease patients may be associated with dysregulation of circadian rhythms, intrinsic 24‐h cycles that control essential physiological functions, or with side effects from levodopa medication and physical and mental health challenges. The impact of circadian dysregulation on sleep disturbances in Parkinson's disease is not fully understood; as such, we review the systems, cellular and molecular mechanisms that may underlie circadian perturbations in Parkinson's disease. We also discuss the potential benefits of chronobiology‐based personalized medicine in the management of Parkinson's disease both in terms of behavioural and pharmacological interventions. We propose that a fuller understanding of circadian clock function may shed important new light on the aetiology and symptomatology of the disease and may allow for improvements in the quality of life for the millions of people with Parkinson's disease.

## Graphical abstract

In Parkinson's disease, pathological α‐synuclein aggregation and Lewy body formation may lead to the death of dopaminergic neurons, which has a bi‐directional influence on circadian rhythm dysfunction and sleep disturbances. L‐dopa treatment and dopamine agonists may also influence circadian rhythm dysfunction. Sleep disturbances such as social jet lag/shift work can have a bi‐directional influence on circadian rhythm dysfunction.

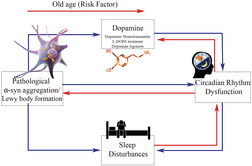

**Abbreviations**
α‐syn: α‐synuclein
6‐OHDA: 6 hydroxydopamine
Aldh2: aldehyde dehydrogenase 2 family member
AD): Alzheimer's disease
Aβ: amyloid‐β
AVP: arginine vasopressin
BMAL1: brain and muscle Arnt‐like protein 1
CK1δ/ε: casein kinase 1 delta and epsilon
CNS: central nervous system
CSF: cerebrospinal fluid
CRY: cryptochrome
DLMO: dim light melatonin onset
EEG: electroencephalogram
EMO: early morning off
EDS: excessive daytime sleepiness
GABA: gamma‐aminobutyric acid
GRP: gastrin‐releasing peptide
ipRGCs: intrinsically photoreceptive retinal ganglion cells
L‐dopa: levodopa
LRRK2: leucine‐rich repeat kinase 2
MPTP: 1‐methyl‐4‐phenyl‐1,2,3,6‐tetrahydropyridine
NF‐κB: nuclear factor kappa B
NLRP3: NOD‐, LRR‐ and pyrin domain‐containing protein 3
NPAS2: neuronal PAS domain protein 2
Nqo1: NAD(P)H quinone dehydrogenase 1
NREM: non‐rapid eye movement
OSA: obstructive sleep apnea
PD: Parkinson's disease
PACAP: pituitary adenylate cyclase‐activating polypeptide
PARK: Parkin
PER: period
REM: rapid eye movement
PET: positron emission tomograph
PINK1: PTEN‐induced kinase 1
RBD: REM sleep behaviour disorder
REM sleep: rapid eye movement sleep
RHT: retinohypothalamic tract
RORα: retinoic acid‐related orphan receptor alpha
SN: substantia nigra
SCN: suprachiasmatic nucleus
TH: tyrosine hydroxylase
TST: total sleep time
VIP: vasoactive intestinal peptide
VTA: ventral tegmental area
Wnt: Wingless‐related integration site

## 1 INTRODUCTION

Parkinson's disease (PD) is the second most common age‐related neurodegenerative disorder of the central nervous system (CNS) (Bekris et al., 2011) and carries a significant and increasing financial and societal global burden; the prevalence of PD has more than doubled over 25 years (1990–2015) (GBD 2015 Neurological Disorders Collaborator Group, 2017). It is estimated that there will be between 8.7 and 9.3 million PD cases in western Europe's five, and the world's 10, most populous nations by 2030 (Dorsey et al., 2007). The progressive loss of dopaminergic neurons in the substantia nigra (SN) resulting from oxidative stress, mitochondrial dysfunction and Lewy body formation are the main pathological mechanisms proposed for PD symptoms (Dexter & Jenner, 2013). PD patients suffer from motor symptoms such as akinesia, bradykinesia, tremor, rigidity and deficits of gait, speech and handwriting (Moustafa et al., 2016) and non‐motor symptoms, which include depression, sleep disturbances, urinary and bowel disturbances and cognitive decline. Both motor and non‐motor symptoms can severely affect the quality of life of patients and caregivers (Corallo et al., 2017; Martínez‐Martín et al., 2005). Sleep disorders have been reported in between 40% and 90% of PD patients and may manifest as somnolence, sleep attacks, rapid eye movement (REM) sleep behaviour disorder (RBD), insomnia and sleep fragmentation (Srinivasan et al., 2014). Many of these sleep disturbances develop as prodromal non‐motor features preceding the motor symptoms for many years or during the symptomatic phase of PD years later (Hustad & Aasly, 2020; Zuzuárregui & During, 2020). There are several potential causes of sleep disturbances in PD patients, including effects of CNS neurodegeneration and neuroinflammation, circadian clock disruptions, ‘classical’ PD motor symptoms leading to poor bed mobility, dopaminergic therapies and co‐morbid psychiatric symptoms (Breen et al., 2014; Chahine et al., 2017; Rye, 2003). In this review, we discuss the evidence for (1) circadian clock disruptions in PD and contrast this with other age‐related neurodegenerative conditions; (2) the relationship between sleep and circadian rhythm disturbances in PD and their consequences for daytime functioning and quality of life; and (3) the application of chronotherapeutic principles for improved PD management strategies.

## 2 THE CIRCADIAN SYSTEM AND SLEEP–WAKE TIMING

Circadian rhythms emerged in evolutionary time as adaptations to the 24‐h light–dark cycle, and these rhythms are primarily generated by the endogenous circadian clock system, which imposes a temporal architecture on cellular, physiological and behavioural processes (Brody, 2013). Circadian rhythms are modulated through exposure to external zeitgebers (a German word meaning ‘time‐giver’), and the dominant zeitgeber for mammals is light; the entrainment of the clock to salient environmental stimuli allows for the maintenance of adaptive rhythms and synchronization of internal time with external cycles (Burke et al., 2015; Farhud & Aryan, 2018; Jensen et al., 2016; Potter et al., 2016). However, circadian clocks are by definition internally generated and will persist in the absence of environmental cycles or time cues (Hastings et al., 2018) and manifest functionally in the temporal regulation of emotional (Correa et al., 2020), cognitive (S. Xu, Akioma, & Yuan, 2021), behavioural (Krylov et al., 2021), metabolic (Serin & Acar Tek, 2019) and endocrine processes (Neumann et al., 2019). Circadian rhythms can be detected at different levels through the cyclic expression of molecular markers, such as clock genes, endocrine markers such as melatonin and cortisol secretion, physiological markers such as core body temperature and behavioural markers such as sleep–wake cycles (Buttgereit et al., 2015).

**FIGURE 1:**
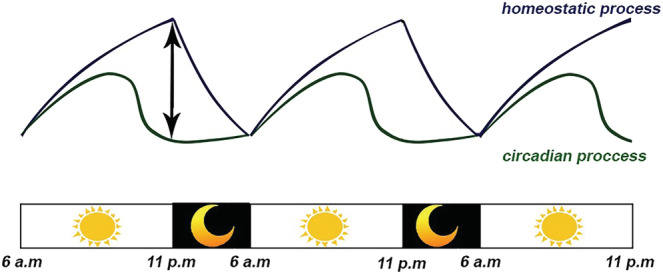
Two process model of sleep in mammals. Sleep is the result of harmony between two processes: The circadian process that relies on the circadian rhythm of light–dark cycles and the homeostatic process that depends on sleep pressure and tiredness. Sleep initiation happens when the distance between sleep pressure and circadian wakefulness is at the maximum level. Following sleep, sleep pressure decreases, and this distance reduces gradually; consequently, wakefulness occurs at the beginning of the next circadian cycle.

Sleep in mammals results from an intricate interplay between two main internal processes, the homeostatic process and the circadian rhythm (Deboer, 2018). The two‐process model for sleep–wake regulation is determined by the accumulation of sleep pressure during the day (process S or homeostatic) and by the circadian process controlled by a 24‐h circadian rhythm (process C [circadian]). The circadian drive for wakefulness reaches its peak in the middle of the day and then gradually wanes until its nadir coincides with the lowest point of core body temperature (Taillard et al., 2021). Sleep initiation happens when the homeostatic sleep pressure is high and the circadian drive for wakefulness is low, leading to a large ‘area under the curve’ drive towards sleep (Figure 1). Continuous interactions between homeostatic and circadian factors, in combination with environmental and social schedules, optimize the rest and activity periods in mammals to meet internal homeostatic demands in the context of an animal's cyclical environment (Arns et al., 2021).

Circadian rhythms depend on intrinsic circadian clocks, circadian input pathways linking the clock to environmental and internal stimuli and signals and output systems at the molecular, cellular and physiological levels (Hastings et al., 2018). The suprachiasmatic nucleus (SCN) serves as the master circadian clock and is in the anterior hypothalamus above the optic chiasma and lateral of the third ventricle (Reppert & Weaver, 2002). There are numerous other circadian oscillators in the CNS that function in a semi‐autonomous form to the SCN, including those in the retina, rodent olfactory bulbs, arcuate nucleus, cerebral cortex, hippocampus and amygdala (Guilding & Piggins, 2007), and the functional consequences of these clocks are believed to modulate cognitive and behavioural processes such as memory, emotional regulation and executive function (Dibner et al., 2010). Clocks are also situated throughout the periphery, including in cellular subtypes in the liver, kidney, heart and pancreas as well as the innate and adaptive immune systems. Across these tissues and systems, circadian clocks regulate cellular, metabolic, endocrine and immune processes and dysregulation of the circadian regulation of such results in various morbidities (Christ et al., 2018).

**FIGURE 2:**
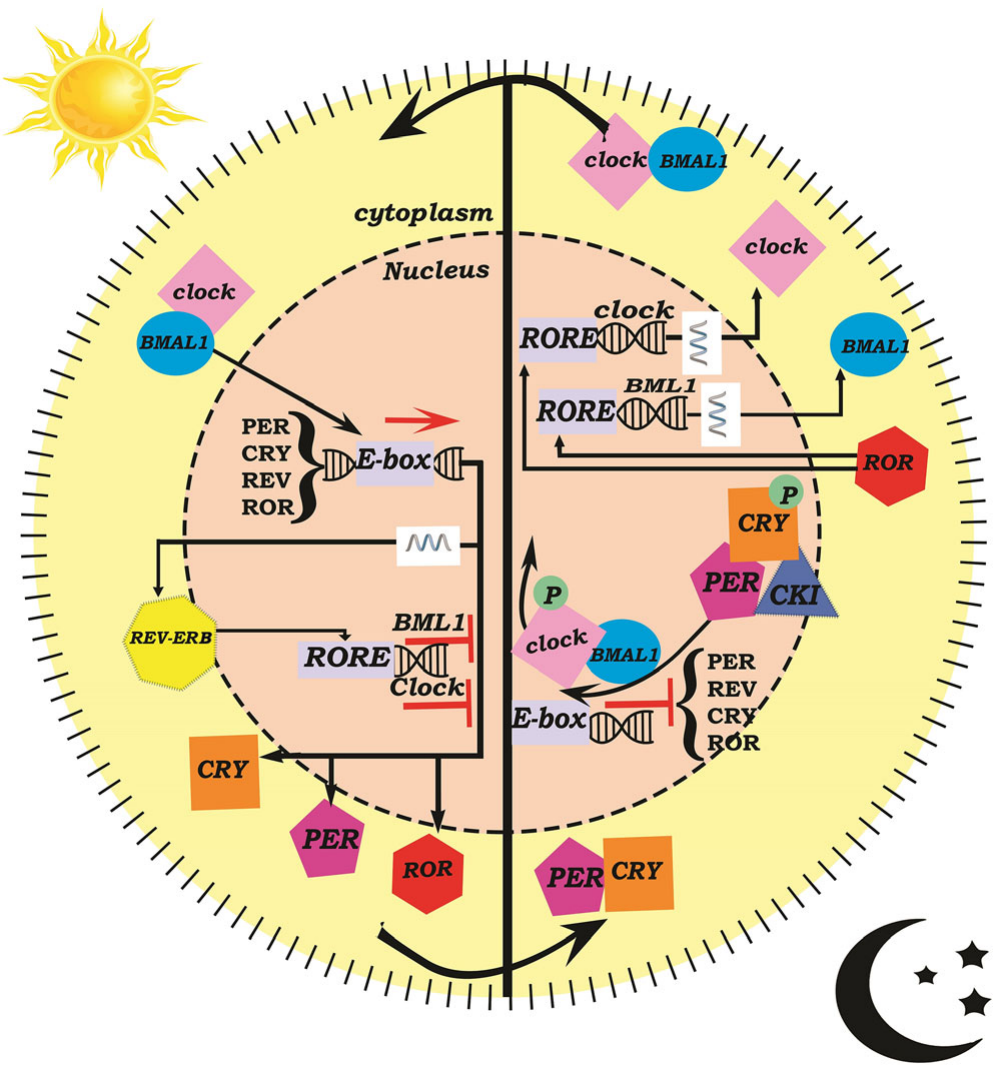
The clock genes network in SCN. CLOCK and BMAL1 dimers activate the E‐box element to initiate the transcription of other clock genes such as PERs, CRYs, RORs and REV‐ERBs. Binding REV‐ERBα to the RORE element of BMAL1 inhibits transcription of this gene. The expression of RORs, PERs and CRYs reaches peak level at 18:00. During the evening, PER:CRY:CK1δ/ε complexes translocate to the nucleus, and CLOCK–BMAL1 dissociates from the E‐box of target genes through CLOCK phosphorylation; consequently, the expression of genes in downstream will be suppressed. At midnight, RORα binds to the RORE of the BMAL1 and CLOCK promoters and induces their transcription. Translation of CLOCK and BMAL1 transcripts during the early morning leads to the initiation of a new cycle. Positive action is indicated by solid lines with lines ending in arrowheads, and negative action is indicated with lines ending in perpendicular lines. BMAL1, brain and muscle Arnt‐like protein 1; CLOCK, circadian locomotor output cycles protein kaput; CRY, cryptochrome; PER, period; REV‐ERBα, nuclear receptor subfamily 1 group D member; ROR, retinoic acid‐related orphan receptor; RORE, retinoic acid‐related orphan receptor response elements

At the molecular level, the circadian rhythms emerge from a molecular network of circadian clocks and their protein products (Kamphuis et al., 2005). The main components in the clock genes network of the circadian system include brain and muscle Arnt‐like protein 1 (*BMAL1*), clock, period (*PER*) 1,2,3, cryptochrome (*CRY*)1,2, neuronal PAS domain protein 2 (*NPAS2*) and *REV‐ERB α* (Mieda, 2019; Takahashi, 2017); these genes encode transcription factors that then form a series of interlocking feedback and feed‐forward loops with periodicities of near 24 h (Figure 2, [Takahashi, 2017]). These circadian clock genes also exert widespread influence over the temporal regulation of the transcriptome; for example, in mice, 40% of transcripts show 24 h cycles in at least one tissue (Zhang et al., 2014). In humans, highly penetrant polymorphisms in clock genes have been implicated in pronounced abnormalities of the sleep–wake cycle (Gentry et al., 2021; Patke et al., 2017).

From a systems perspective, whole‐organism circadian timekeeping results from a hierarchical and distributed system with the SCN at the top of this multi‐oscillator hierarchy (Dibner et al., 2010). SCN neurons have autonomous rhythmic electrical and metabolic processes, even in the absence of neural inputs in dispersed in vitro settings, and these neurons communicate with other regions in the brain through both neuronal and humoral pathways (Shirakawa et al., 2000; Tousson & Meissl, 2004; Welsh et al., 1995). The majority of SCN neurons are GABAergic, and subsets of SCN neurons express neuropeptides such as arginine vasopressin (AVP), vasoactive intestinal peptide (VIP) and gastrin‐releasing peptide (GRP) (Ono et al., 2021). Additionally, glial cells contribute to circadian function in the SCN through their own intrinsic clocks and by modulating neurotransmission (Brancaccio et al., 2019). The SCN receives afferent inputs from several pathways, the most significant of which is the retinohypothalamic tract (RHT). The RHT conveys photic information to the SCN and arises from a subset of intrinsically photosensitive retinal ganglion cells, which express the photopigment melanopsin, and RHT terminals release glutamate and pituitary adenylate cyclase‐activating polypeptide (PACAP) onto the photorecipient areas of the SCN (Hughes et al., 2015). The exact mechanisms of how the SCN produces organism‐level circadian rhythmicity and harmonises the behavioural and physiological cycles (such as sleep, endocrine secretion and body temperature) are not fully understood. However, it is known that the primary recipients of SCN projections are other hypothalamic areas and the midline thalamus (Li et al., 2012).

## 3 A FRAMEWORK FOR UNDERSTANDING CIRCADIAN RHYTHM DYSFUNCTION IN PARKINSON'S DISEASE

In order to consider the evidence linking sleep disturbances and disorders, circadian rhythm dysfunction and motor and other non‐motor symptoms of PD, we will discuss the evidence that (1) older age and other age‐related neurodegenerative conditions are associated with sleep and circadian rhythm changes; (2) PD is associated with sleep problems, and the relationship of such problems to PD symptom type and severity; (3) PD is associated with dysfunction of the circadian system, and the interplay between such dysfunction and other symptoms of PD; (4) the bidirectional relationships between the circadian and dopaminergic systems, and the implications of these relationships for understanding circadian dysfunction in PD; (5) what preclinical/animal models of PD reveal about the relationships between circadian function and PD; and (6) how such insights can be leveraged for the improvement in the prevention and management of PD and the research agenda that will be needed to enable this.

## 4 CIRCADIAN RHYTHMS IN OLDER AGE AND NEURODEGENERATION

As PD is primarily a disease of older age, appreciating the impact of healthy ageing on the circadian clock is of importance in the elucidation of the impact of PD on the clock. Healthy ageing results in changes in circadian function in older adults, including reduced circadian rhythm amplitude in sleep–wake cycles, advanced circadian phase and earlier chronotype, more inter‐daily variability in activity cycles and altered circadian responses to light (Duncan, 2020; Popa‐Wagner et al., 2017). Older age is also associated with other changes in sleep such as reduced sleep efficiency, lower total sleep time, reduced REM sleep time, greater sleep onset latency, periodic limb movement and increased arousal index (Garbarino et al., 2021). Some of these changes may be linked to cellular and systemic changes in the circadian network; for example, ageing may be associated with a higher rate of cell loss in the SCN and pineal gland (Khuzhakhmetova et al., 2019), decreased neuroplasticity and altered neurophysiology of the SCN neuronal network (Buijink et al., 2020; Nakamura et al., 2011), and loss of photoreceptive retinal ganglion cells (ipRGCs) in the eye and their reduced projections to the SCN (Buijink & Michel, 2021). Levels of nocturnal melatonin are also decreased in older age (Godfrey et al., 2022), and this decrease may be linked to age‐related calcification of the pineal gland (Bumb et al., 2014). As such, ageing is associated with alteration to input pathways to the master circadian clock, core clock function and output pathways of the master clock that disseminate the clock's temporal signal throughout the body.

It is of interest to examine the circadian and sleep changes associated with other neurodegenerative conditions to appreciate transdiagnostic features of sleep and rhythm disturbances across such conditions vs diagnosis‐specific changes. Circadian rhythm changes observed in age‐related neurodegenerative conditions, such as Alzheimer's disease (AD), are found to be markedly more pronounced than those observed in healthy ageing (Coogan et al., 2013; Hunt et al., 2022). Circadian function is found to be progressively impaired in AD, with a marked decrease in the amplitude of the sleep–wake cycle being a prominent feature of a moderate‐to‐severe disease (Coogan et al., 2013). Although AD is not associated with large‐scale neurodegeneration in the SCN (Fernandez et al., 2021), there may be a loss of specific neurochemical neuronal phenotypes in the SCN, such as cells expressing neuropeptides strongly implicated in normal circadian function such as neurotensin, VIP and AVP, reduced expression of the MT1 melatonin receptor and reduced GABA signalling, dysregulated redox in the SCN and reduced BMAL1 expression in activated astrocytes of SCN (Singer & Alia, 2022). As such, the integrity of the SCN neuronal network may be reduced and circadian timing impacted through this. Outside of the SCN, other neurochemical and neuropathological changes in AD that have been linked to declined circadian and sleep function are reduced expression of orexin A and adenosine A1 receptor (Liu et al., 2019), degeneration and atrophy of cholinergic neurons and cholinergic nuclei in the basal forebrain and degeneration of hypothalamic orexin neurons (Matsumoto & Tsunematsu, 2021).

Although the exact mechanistic link between circadian rhythms disruption and neurodegeneration is not clear, it has been reported that dysregulation in circadian rhythmicity drives astrocyte and microglial activation and polarization, activation of NFκB and NLRP3 inflammasome mediated inflammation pathways in the CNS, lower levels of expression in redox defence‐related genes like *Nqo1* and *Aldh2* in the CNS and increases in α‐synuclein aggregation (Griffin et al., 2019; Huang et al., 2018; Kou et al., 2022; Lananna et al., 2018; Liu et al., 2022; Musiek, 2015). Results from several studies suggest a bi‐directional relationship between neurodegeneration and circadian rhythms (Colwell, 2021): neurodegeneration leads to circadian dysregulation, and dysregulated circadian processes contribute to neurodegeneration (Nassan & Videnovic, 2022). For instance, amyloid‐β (Aβ) accumulation in AD leads to sleep complaints, and sleep disorders increase the risk of amyloid deposition and dementia development in affected individuals (Ju et al., 2014). It has been hypothesized that there is an age effect on the association between sleep problems and Aβ accumulation, as both intensify with age and may interact to result in increased neuropathology and cognitive and behavioural decline (Chong et al., 2022). There is evidence from pre‐clinical models that long‐lasting neuroinflammation in the absence of neurodegeneration can induce persistent circadian changes (O'Callaghan et al., 2012), that geriatric microglia have been implicated in age‐relate declines in sleep quality (Choudhury et al., 2021) and that decreased circadian rhythmicity in microglia is implicated in age‐related neuroinflammation (Fonken et al., 2016); all of these features suggest that neuroinflammation may be an important link between the circadian clock and neurodegenerative disorders.

**FIGURE 3:**
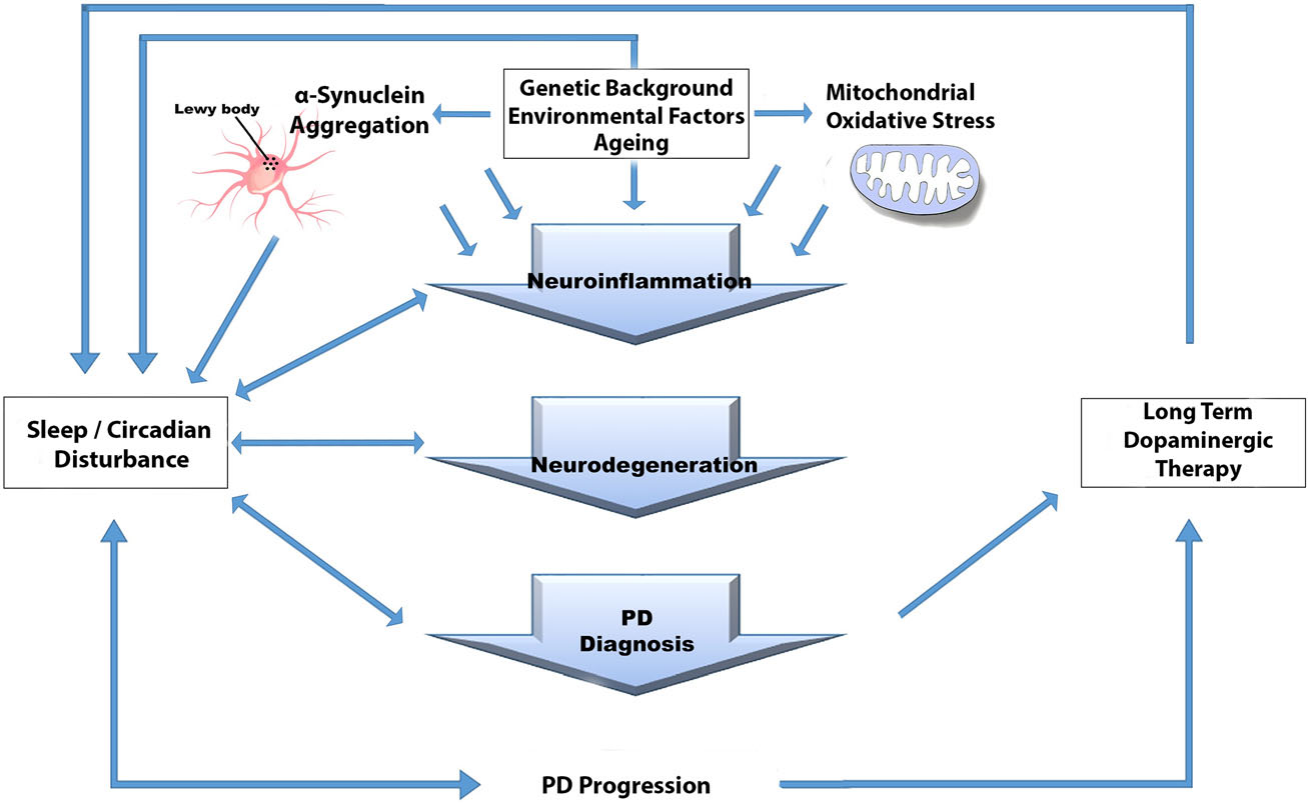
A bidirectional relationship between circadian/sleep disturbances and PD diagnosis/progression. Neuroinflammation and neurodegeneration in dopaminergic neurons, originating from various genetic and environmental factors, lead to PD incidence. It seems that circadian and sleep alteration is not only an early symptom of PD but also an etiologic factor for PD occurrence. Furthermore, there is an association between sleep and circadian rhythm function with exacerbation of PD symptoms. There is also some evidence that long‐term L‐Dopa treatment may accelerate sleep and circadian rhythm manifestations in PD patients. PD, Parkinson's disease

Of interest when considering sleep and circadian rhythm changes in PD, sleep disturbances are common in the prodromal stages of AD (Duncan et al., 2021). Impaired circadian rhythmicity may be both an early symptom of neurodegenerative diseases and also a risk factor for the subsequent development of neurodegenerative conditions (Kumar et al., 2022). Some studies have also reported that lower circadian rhythmicity is associated with greater cognitive decline in older adults who do not have a diagnosis of dementia, although more investigation is required to determine the aetiology and significance of such findings (Cochrane et al., 2012; Rogers‐Soeder et al., 2018; Walsh et al., 2014). Given that dementia is common in PD patients (Goetz, 2009), the emerging literature on sleep and circadian factors as early prodromal markers and/or risk factors for AD suggests that future focus on such issues in PD may identify novel risk factors and opportunities for intervention (Figure 3; Lananna & Musiek, 2020; Nassan & Videnovic, 2022; Leng et al., 2019).

## 5 SLEEP DISORDERS AND DISTURBANCES IN PARKINSON'S DISEASE

A variety of sleep disorders and disturbances are observed in PD patients including, RBD, insomnia disorder, obstructive sleep apnea (OSA), sleep fragmentation, decreases in the quantity of non‐rapid eye movement (NREM) sleep, low sleep efficiency and excessive daytime sleepiness (EDS) (Comella, 2006; Stavitsky et al., 2012). These sleep problems affect the quality of life in PD patients in both prodromal and symptomatic stages of the disease, with patients with poorer quality sleep experiencing more severe motor and non‐motor symptoms (Al‐Qassabi et al., 2017). Post‐mortem analysis of PD patients' brains has revealed that α‐synuclein pathology and neuronal loss in some brain's regions are associated with sleep alterations in PD (Braak et al., 2004; Thannickal et al., 2007). However, as sleep alterations are present during the prodromal stage of PD, possibly without pathologically significant neuronal loss, sleep alterations may become manifest at levels of neurodegeneration below the threshold to cause classic PD motor symptoms and clinical diagnosis. There is a reported association between sleep fragmentation and Lewy body pathology and substantia nigra degeneration in patients without a clinical diagnosis of PD, indicating a potential for a more generalised link between neurodegeneration and neuropathology with sleep problems independently of classic features of parkinsonism and that sleep fragmentation may represent a risk factor for progression to PD (Sohail et al., 2017).

Interestingly, aside from neurodegeneration in the substantia nigra, loss of non‐dopaminergic neurons, such as hypocretin, norepinephrine and serotonin‐releasing neurons, is also associated with sleep disturbances in PD patients. For example, the early onset of narcolepsy‐like sleep manifestations, such as frequent wakening and daytime sleepiness, in PD patients may originate partially from loss of hypocretin neurons in the hypothalamus, and the rate of loss of hypocretin neurons increases with advancing Braak staging of PD neuropathology (Braak et al., 2003; Thannickal et al., 2007). The results of a recent study using positron emission tomography (PET) scanning with a highly selective radioligand for the noradrenergic transporter revealed that the density of noradrenaline transporter decreases in arousal prompting locus coeruleus and raphe nuclei in PD patients (Doppler et al., 2021); both these midbrain regions are known to play key roles in homeostatic sleep regulation and vigilance state setting (Osorio‐Forero et al., 2022), and as such, their perturbation in PD may be of particular significance for understanding sleep problems in the disease. In addition, the results of PET scanning in PD patients highlight the potential roles of serotonergic neurons in sleep disorders in PD patients, with some regions that are critical in sleep regulation, such as the ventral striatum, thalamus, hypothalamus, raphe nuclei, caudate and putamen showing lower expression of serotonin transporter in PD patients with sleep disturbances (Wilson et al., 2018).

RBD is a sleep disorder characterised by a failure of muscle atonia during REM resulting in dream enactment and other sleep behaviours (Schütz et al., 2022). RBD is a common early sleep complaint in neurodegenerative α‐synucleinopathy diseases (Boeve et al., 2001) and is reported in up to 60% of PD patients; its prevalence is associated with age, male sex, disease severity, duration of motor symptoms and dopaminergic therapy (Lee et al., 2010; Zhu et al., 2017). The result of a multi‐centre study has indicated that RBD is a prodromal marker for PD, with 73% of RBD patients being diagnosed with neurodegenerative syndromes within 12 years of follow‐up, and 57% of the patients developed Parkinsonism as the first disease symptom (Postuma et al., 2019). RBD is also a prognostic factor for PD outcome; patients with RBD have poorer prognoses in motor and non‐motor symptoms, autonomic functions and mortality rate compared with non‐RBD patients (Kim et al., 2018; Romenets et al., 2012). Postulated reasons for the association between RBD and PD severity include the suggestion that the underlying neurodegeneration patterns in patients with or without RBD are different, with RBD being associated with the shrinkage of critical brain regions such as the pontomesencephalic tegmentum, hypothalamus, thalamus, medullary reticular formation, anterior cingulate cortex, putamen and amygdala (Boucetta et al., 2016). It has recently been suggested that the circadian system influences the brainstem circuits implicated in RBD, and as such, the circadian clock may represent a novel therapeutic target for RBD (Venner et al., 2019). Further, recent evidence has suggested that the circadian regulation of nocturnal dipping of blood pressure is disrupted in RBD (Terzaghi et al., 2022) and that RBD is associated with reduced robustness of the diurnal sleep–wake cycle (Liguori et al., 2021). These findings suggest that RBD may be associated with core deficits in circadian function, although further work is clearly required to better understand the circadian contributions and consequences of RBD.

Insomnia disorder is characterised by having difficulty falling asleep or staying asleep or experiencing unrefreshing sleep over a sustained period and has a reported prevalence of approximately 10% in the general adult population (Riemann et al., 2022). Insomnia disorder is reported in 55% of PD patients (Sobreira‐Neto et al., 2017), with associated risk factors including female gender, PD duration, anxiety, depression, circadian dysfunction, inadequate use of medications and medication adverse effects (Gros & Videnovic, 2017; Mizrahi‐Kliger et al., 2022). The frequency of sleep–wake disturbances in PD progressively increases over time, and insomnia is the most frequent sleep complaint in PD at any given time (Z. Xu, Anderson, et al., 2021). PD patients with insomnia disorder have longer REM sleep onset latency compared to those without, suggesting problems relating to sleep initiation (Sobreira‐Neto et al., 2020). Associations between insomnia symptoms and severity of motor symptoms in PD are reported to vary from positive associations (Caap‐Ahlgren & Dehlin, 2001) to no association (Chung et al., 2013), but insomnia symptoms severity clearly associate with other non‐motor symptoms, such as mood disorders, in PD (S. Xu, Akioma, & Yuan, 2021). Circadian clocks have been strongly implicated in the aetiology and symptomatology of insomnia disorder (Nobre et al., 2021), and as such, circadian dysfunction in PD may be an important contributor to the high level of insomnia reported in PD.

OSA is a common sleep disorder in the general population (~20% according to the apnea–hypopnea index) and is characterised by recurring instances of the collapse of the upper airway during sleep leading to apnea and hypopnea (Franklin & Lindberg, 2015). OSA has a high prevalence in PD, with 62% of patients with PD reported fulfilling the diagnostic criteria (Kaminska et al., 2015). Risk factors for OSA in PD include high body mass index, older age and being male (Dziewas et al., 2008). In the general population, insomnia disorder and OSA are frequently comorbid, and this may well be the case in PD (Cruz et al., 2021). OSA is described as having a strong circadian component in its aetiology (von Allmen et al., 2018), and as such, understanding the roles of circadian rhythms in PD may contribute to the understanding of the high prevalence of OSA in PD, as well as its co‐morbidity with insomnia disorder.

EDS, which presents as difficulty staying awake or alert and an increased tendency to fall asleep during the day, is a common complaint in PD, being reported by 20%–60% of PD patients (Chahine et al., 2017). EDS in PD is associated with greater age, male gender, postural instability and gait disorder, depressive symptoms, cognitive impairment, anxiety, sleep fragmentation at night, levodopa (L‐Dopa) equivalent daily dosage and pain (Borek et al., 2006; Feng et al., 2021; Gera & Comella, 2022; Höglund et al., 2015, 2019). EDS is associated with nigrostriatal degeneration in PD but may also be associated with dopamine agonist treatments (Gjerstad et al., 2006; Happe et al., 2007; Paus et al., 2003; Prudon et al., 2014). EDS risk and severity in PD patients increase over time, with prevalence in PD being reported to increase from 5.6% at baseline to 41% after 8 years of follow‐up (Gjerstad et al., 2006). PD patients with EDS have more severe motor and non‐motor PD symptoms and lower quality of sleep (Xiang et al., 2019), lower quality of life, more impaired daily functioning and increased burden of disease (Sobreira‐Neto et al., 2017). EDS is linked with circadian function, possibly through a reduced circadian drive to sleep during the night and to arousal during the day (Gandhi et al., 2021). EDS is also strongly associated with insomnia disorder and OSA and so may be prominent in PD as a result of co‐morbid sleep problems.

## 6 CIRCADIAN RHYTHMS IN PARKINSON'S DISEASE

Findings from studies using wrist‐worn actigraphy indicate the presence of changes in the daily rhythm of sleep–wake cycles in PD patients compared to age‐matched controls. Obayashi et al. (2021) show a phase advance of the sleep–wake cycle in later stage PD and a decrease in the amplitude of the rhythm due to the presence of greater nocturnal activity and lower daytime activity. Brooks et al. (2020) report similar findings, highlighting the importance of clinical staging in sleep–wake alterations in PD and the usefulness of actigraphy in the identification of such changes. More variability in the day‐to‐day sleep–wake rhythm in PD was found to be associated with poorer executive, visuospatial and psychomotor functions (Wu et al., 2018). Actigraphy has also revealed that RBD is associated with sleep–wake rhythm fragmentation, and these changes serve as predictive indicators of clinically diagnosed α‐synucleinopathy (Feng et al., 2020). Further, Leng et al. (2020) describe that patients at an elevated risk of developing PD show lower circadian rhythm amplitude and robustness in the sleep–wake cycle but did not show changes in acrophase of the rhythm. As such, it may be that PD is associated primarily with fragmentation and increased variability of the daily sleep–wake rhythm and that later stage disease is associated with an altered circadian phase. In terms of rhythms in physiological processes, PD patients have been reported to experience circadian rhythm dysregulations manifested in alterations in diurnal profiles of blood pressure (71% of PD patients do not express rhythms; Berganzo et al., 2013).

In the discussion and appraisal of findings from actigraphy in PD patients, it should be noted that these devices and their analytical algorithms are often only validated in healthy individuals, and it is often unclear as to how their validity translates to studies in patients with movement disorders and other symptoms that impact on motor activity (Xue et al., 2022). It has been shown that actigraphy can be usefully used in PD and validated against ‘gold‐standard’ polysomnography, although it is not clear which adjustment to the scoring algorithm is optimal for accuracy and specificity and that there is a high level of inter‐patient variability comparing autographic scores to polysomnography outcomes (Maglione et al., 2013). The placement of the actigraphy device between dominant and non‐dominant arms does not appear to exert an important effect on outcomes in PD, although there were differences between upper limb and lower limb device placement (Prasad & Brown, 2017). Other studies have sought to pilot novel ambulatory multi‐sensory devices for sleep monitoring in PD, and such approaches may ultimately provide enhanced sensitivity above traditional actigraphy (Madrid‐Navarro et al., 2019). At present, findings in PD from actigraphy should be interpreted with the caveat of the limited validation of such approaches in the specific context of PD.

Polymorphisms in clock genes have been associated with PD incidence (D'Alessandro et al., 2017; Gu et al., 2015; Zou et al., 2020). Single‐nucleotide polymorphisms purported to be associated with PD include ones in the clock genes *CLOCK*; *BMAL‐1*; *PER1,2*; and *CRY1,2*, and it has been proposed that alterations in clock genes might contribute to PD pathogenesis through alteration of circadian modulation of process such as mitochondrial bioenergetics, autophagy and alteration in neuroendocrine function (Shkodina et al., 2022). Dysregulation in the expression of circadian genes has been indicated in PD patients by several studies using approaches such as expression in peripheral blood mononuclear cells or ex vivo approaches utilizing assays in primary fibroblasts derived from PD patients (Breen et al., 2014; Cai et al., 2010; Pacelli et al., 2019). A negative association has been reported between the strength of the rhythmic expression of clock genes and RBD and/or excessive daytime sleepiness in PD patients (Li et al., 2021). There is also a reported positive correlation between the nocturnal expression of *BMAL1* and PD symptom severity (Cai et al., 2010). The precise nature, mechanisms and consequences of alterations in the expression of clock genes in PD are far from well elucidated, and factors such as altered epigenetic regulation of clock genes in PD may play a role in such changes (Lin et al., 2012; Liu et al., 2008).

Circadian rhythm dysregulation reported in PD is also manifest in the timing of rhythmic secretion of cortisol and melatonin and core body temperature rhythms (Raupach et al., 2020; Zhong et al., 2013). PD patients were reported to have arrhythmic cortisol profiles or later cortisol rhythm acrophase and greater area under the curve of cortisol (Breen et al., 2014; Soares et al., 2019). For melatonin, PD patients were reported to have lower rhythm amplitude and area‐under‐curve (Videnovic et al., 2014) and lower secretion levels (Breen et al., 2014; Sanchez‐Barcelo et al., 2017; however, a higher plasma melatonin level [Li et al., 2020] and a blunted peak of the rhythm [Zuzuárregui & During, 2020] have also been reported). Furthermore, there are reported associations between melatonin levels and PD severity, with increasing symptom severity or hypothalamic volume associated with more alterations in melatonin secretion profiles (Breen et al., 2016; Li et al., 2020). Pineal gland calcification, loss of hypothalamic grey matter and lower density of MT1 and MT2 receptors in the amygdala and substantia nigra have been suggested to associate with altered melatonin output and sleep disturbance in PD (Adi et al., 2010). A correlation between excessive daytime sleepiness and lower nocturnal serum melatonin has also been reported (Uysal et al., 2018).

Assessment of dim light melatonin onset (DLMO), a biomarker for the circadian clock phase, in PD patients has demonstrated that the phase angle of entrainment (the discrepancy between circadian phase and sleep/activity onset) is larger in PD patients on medication compared to PD un‐medicated patients, indicating a role for PD medication in alterations of melatonin rhythms and the relationship between the circadian and the sleep–wake cycles, in that dopaminergic therapy was associated with delayed sleep onset relative to the circadian phase as estimated by DLMO (Bolitho et al., 2014). Alterations in melatonin rhythmicity are also implicated in RBD in PD (Weissová et al., 2018): Although some studies have failed to observe a significant difference in the 24‐h melatonin rhythm between RBD patients and healthy controls (Breen et al., 2014), other results indicate that melatonin rhythmicity differs between PD/RBD patients and controls in that the melatonin rhythm is delayed by approximately 2 h, in parallel with a 1 h delay in habitual sleep phase (Weissová et al., 2018). These findings suggest that previously reported inconsistent findings regarding alteration in the circadian phase in PD as derived from DLMO or core body measurements (Bolitho et al., 2014; Videnovic et al., 2014; Zhong et al., 2021) may be partly due to differing clinical characteristics of the cohorts examined in terms of medication status, disease severity and presence of RBD.

## 7 CIRCADIAN RHYTHMS, DOPAMINE AND DOPAMINERGIC THERAPY

As degeneration of substantia nigra dopaminergic neurons is primarily implicated in the pathophysiology of PD, it is important to understand the links between the circadian system, dopamine and PD. In considering such relationships, there is a strong potential for bidirectionality: PD‐related loss of dopaminergic neurons may alter core circadian function, whereas circadian rhythm disruption may increase PD risk (Leng et al., 2020). Further, as frontline pharmacotherapy for PD involves drugs that act on the dopaminergic system, it is of clinical importance to appreciate how such treatments may influence, and be influenced by, the circadian system. Further, homeostatic sleep changes related to neurodegeneration in PD and PD drug treatment may themselves interact with circadian processes to indirectly produce circadian rhythm abnormalities (Borbély et al., 2016). Although it is beyond the scope of the current review to overview all of the circadian dopamine, below, we will focus on some of the facets of these relationships that may be most pertinent for PD.

Dopamine exerts a direct effect on circadian rhythm regulation as dopamine receptors are expressed widely within the SCN, and midbrain dopaminergic neurons innervate the SCN and can induce phase shifts of SCN‐driven behavioural rhythms and mice null for the dopamine receptor Drd1 show altered photic entrainment of their circadian rhythms in an SCN‐dependent manner (Grippo et al., 2017). Treatment of the SCN in vitro/ex vivo with dopamine or dopaminergic agonists can induce circadian changes (Grippo & Güler, 2019), and dopamine treatment can also alter the rhythmic expression of clock genes outside of the SCN; for example, dopamine treatment decreases *PER1* and *PER2* rhythmicity as well as *CRY1* and *PER* genes expression in human dermal fibroblast cultures (Faltraco et al., 2021). As such, there are reasonably strong indicators that dopamine can alter behavioural and molecular circadian processes, and as such, the loss of dopaminergic signalling in PD may directly contribute to alterations in circadian rhythms.

The next issue is to appreciate how the circadian system may influence dopamine signalling and how such processes may be related to PD pathogenesis/physiology. Substantia nigra slice cultures display rhythmic expression of *per2*, indicating the presence of at least a semi‐autonomous circadian clock in the SNc (Natsubori et al., 2014). Dopamine biosynthesis displays circadian rhythmicity through the cyclic expression of tyrosine hydroxylase (*th*), which is regulated by competition between *rev‐erbα* and nuclear receptor‐related 1 protein (*nurr1*) (Chung et al., 2014; Kim et al., 2017). Dopamine has two main types of G‐proteins coupled receptors: Gs‐coupled (D1 and D5) and Gi‐coupled receptors (D2, D3 and D4) (Beaulieu & Gainetdinov, 2011). In addition to the diurnal rhythm in dopamine release (Poceta et al., 2009), dopamine receptors also may have a circadian rhythm that relies mostly on the site of receptor expression. For example, the D3 receptor has a circadian variation in the ventral striatum (Myslivecek, 2021). As such, there are a number of points in the nigrostriatal pathway that may operate under control by the circadian clock. There are strong indications that the mesolimbic, mesocortical and other dopaminergic pathways are strongly influenced by the circadian system (Pradel et al., 2022), and such influences may be important in the consideration of conditions such as schizophrenia (Ashton & Jagannath, 2020).

PD treatment is primarily aimed at symptomatic relief, with the mainstay of clinical management of PD being L‐Dopa usually in combination with an inhibitor of aromatic amino acid decarboxylase enzyme, which reduces the required L‐dopa dose. Treatment regimens are titrated to disease stage, symptom severity and individual patient profile (Ahlskog, 2011; Pezzoli & Zini, 2010). L‐Dopa is absorbed in the small intestine, taken up by striatal terminals from the substantia nigra and converted into dopamine by intraneuronal enzymes (Pezzoli & Zini, 2010). Oral dopamine agonists such as pramipexole and ropinirole are associated with risks of sedation and sleep attacks (Frucht et al., 1999); therefore, they are not recommended in PD patients with sleep–wake disorders. It has been observed that other drug treatments that target the dopaminergic system, such as antipsychotics, impact circadian processes (Coogan et al., 2011), and dopamine also impacts circadian rhythms (Faltraco et al., 2021); such findings provide a context to appreciate the impact of pharmaceutical drug therapies on circadian processes in PD.

Some investigations of L‐Dopa's impact on circadian rhythms in PD have revealed that PD patients with L‐Dopa treatment display an advanced circadian phase (Bordet et al., 2003; Martino et al., 2018) and levodopa treatment may be an important contributor to circadian rhythm disruptions in PD (Figure 3, Liu et al., 2021). L‐Dopa treatment is strongly associated with sleep complaints such as insomnia, daytime somnolence, vivid dreams, nocturnal vocalisation and myoclonus (Nausieda et al., 1982). The timing of treatment may be important as it has been demonstrated that administration of levodopa at night significantly delays its absorption rate and reduces the maximum concentrations of levodopa at night, suggesting that the circadian system has a strong effect on gastric emptying and consequently the absorption rate of L‐Dopa (Nyholm et al., 2010). From pre‐clinical studies, L‐Dopa administration once daily at 15:00 for 21 consecutive days in an animal model of PD (6‐OHDA‐treated rats) improves motor deficiencies but also leads to phase delays and a lower amplitude in the expression of clock genes in the SCN and striatum whilst also eliminating the rhythm of striatal dopamine and increasing the average amount of dopamine in the striatum (Li et al., 2017). Conversely, there is some evidence that lower L‐Dopa metabolites in cerebrospinal fluid (CSF) are associated with sleep complaints (Chong et al., 2022). Thus, the dose and timing of L‐Dopa administration could possibly be optimized based on chronobiology to mitigate undesirable effects on circadian rhythms and sleep.

An important aspect of L‐Dopa therapy is the ‘early morning off (EMO)’ effect, wherein symptoms recur whilst medication is wearing off in the morning and occurs in more than 50% of PD patients receiving dopaminergic treatment (Han et al., 2020). Delay in time to ON (the symptoms improved after taking the medication) is the strongest predictor of motor function fluctuations in PD patients using L‐Dopa treatment (Han et al., 2020; Stocchi et al., 2019). Furthermore, non‐motor symptoms fluctuations including pain, paresthesia, akathisia, tightening, tingling sensations and sensory dyspnea are frequent in PD patients with both longer duration of disease onset and higher L‐Dopa dose (Bayulkem & Lopez, 2011; Brun et al., 2014; Raudino, 2001; Witjas et al., 2002). Appreciating how circadian and sleep factors influence L‐Dopa pharmacokinetics and therapeutic actions may be helpful in identifying patients at the greatest risk for EMO and mitigating such risks. For example, there is a reported association between higher sleep efficiency and lower sleep fragmentation with shorter early‐morning akinesia, an association that is independent of confounding factors such as age, disease stage, daily activity level, sleep medications, levodopa equivalent dose and RBD symptoms (Kataoka et al., 2020). It may be that the effect of better sleep quality and a subsequent reduction of morning akinesia is related to the upregulation of dopamine D2 receptors and a reduction in dopamine release during night‐time, allowing for more dopamine release in the morning leading to lower morning akinesia (Kalia & Lang, 2015). Conversely, if L‐Dopa levels decline during the night, low morning dopamine levels and EMO may occur (Swope, 2004). Thus, circadian rhythm regulation, good quality sleep and appropriate timing of L‐Dopa administration may reduce the incidence of EMO.

If L‐Dopa treatment is associated with sleep and circadian rhythm disturbance, then chronotherapy, such as the use of light therapy and/or melatonin treatment, may be useful treatment adjunct to minimise such effects (Fifel & Videnovic, 2019; Smolensky et al., 2016). Light therapy in PD patients receiving L‐dopa treatment was reported to improve sleep, motor function and depression indicating that part of the circadian dysfunction in PD may be due to pharmacotherapy (Endo et al., 2020; Sun et al., 2022). Indeed, evidence that such an effect arises from the amelioration of circadian dysfunction is supported by findings of rapid attenuation of oscillations in in vitro clock genes expression in mouse immortalized SCN neurons following chronic dopaminergic treatment (Endo et al., 2020). Further investigation of the potential of chronotherapy for the alleviation of disease‐and‐treatment‐related sleep and motor symptoms in PD is warranted as the current evidence base may be of sufficient quality to draw clinically useful conclusions (Huang et al., 2021).

## 8 CIRCADIAN RHYTHMS IN ANIMAL MODELS OF PD

Conducting circadian rhythm research in human populations brings many challenges, including achieving adequate statistical power, clinical heterogeneity of study samples, limitations on the period of sleep–wake monitoring and the high demand characteristics for sampling regimes required for assays of rhythmic biomarkers, environmental variables and social factors and transcultural differences in daily schedules and norms and difficulties in attaining mechanistic insights or establishing causality in relationships (Blatter & Cajochen, 2007). As such, the coordination of clinical studies and those utilizing pre‐clinical models has significant potential to advance the understanding of the interplay of PD with the circadian system and sleep (Hunt et al., 2022; Mizrahi‐Kliger et al., 2022). Pre‐clinical investigations allow for tight control of environmental and genetic factors, in‐depth circadian phenotyping at the behavioural, physiological and molecular levels, relative ease of longitudinal analysis and assessment of pharmacological, environmental and behavioural interventions that would be highly challenging in human studies. In chronobiology research, the use of pre‐clinical models has been essential to the field and has led to important translation to human health science; for example, the 2017 Nobel Prize for Physiology or Medicine was awarded for fundamental work on the genetic basis of the circadian clock in *Drosophila Melanogaster* that translated to the understanding of the genetic basis of familiar circadian rhythm sleep disorders (Huang, 2018). As such, there is burgeoning interest in the use of animal models for investigating sleep and circadian function in PD (Table 1; [Hunt et al., 2022]).

**TABLE 1:** Circadian rhythm changes in animal models of PD

Model	Intervention	Species	Circadian phenotype
Genetic based models	PARK & PINK1 mutant	*Drosophila Melanogaster*	Greater sleep fragmentation and lower circadian power (Valadas et al., 2018)
	LRRK2 mutant	Mouse	Lower *Clock* expression and reduced REM, NREM and total sleep time with an increased wake time in transgenic mice compared to control (Liu et al., 2022)
	α‐SYN overexpression	Mouse	Increased sleep onset latency, lower power in circadian rhythm (Kudo et al., 2011), increased NREM sleep, decreased REM sleep and altered oscillatory EEG activity (McDowell et al., 2014)
	Mutant α‐SYN (A53T)	Mouse	Reduced NREM sleep and reduced total sleep time (Peters et al., 2020)
	MitoPark	Mouse	Increased sleep onset latency (Langley et al., 2021) and reduced circadian rhythm amplitude and stability (Fifel & Cooper, 2014; Peters et al., 2020)
Neurotoxin based models	MPTP	Mouse	Lengthened free‐running period (Tanaka et al., 2012), alterations of clock genes expression and reduced amplitude of circadian rhythm in locomotor activities (Hayashi et al., 2013)
		Non‐human primates	Sleep alterations during the day and night (Choudhury & Daadi, 2018), loss of rhythmic locomotor outputs without environmental cues (Fifel et al., 2014) and no alteration in circadian rhythm (Franke et al., 2016)
	6‐OHDA	Rat	Alterations in clock genes expression (Li et al., 2017; Wang et al., 2018), in parameters of locomotor circadian rhythm (Souza et al., 2018), and in circadian rhythms of blood pressure and body temperature (Yang et al., 2021)
	Rotenone	Rat	Alterations in clock genes expression (Mattam & Jagota, 2015), reduced rhythm amplitudes and increased fragmentation in rhythm (Lax et al., 2012)

Abbreviations: α‐SYN, α‐synuclein; 6‐OHDA, 6‐hydroxydopamine; EEG, electroencephalogram; LRRK2, leucine‐rich repeat kinase 2; NREM, non‐rapid eye movement; MPTP, 1‐methyl‐4‐phenyl‐1,2,3,6‐tetrahydropyridine; PARK, Parkin; PINK1, PTEN‐induced kinase 1; REM sleep, rapid eye movement sleep; TST, total sleep time.

Studies on a transgenic mouse model of PD that overexpressed the SNCA gene that encodes α‐synuclein demonstrated some alterations in sleep and circadian rhythm parameters, including a reduction in the firing rate of SCN neurons, dampened circadian rhythms with ageing and disease progression and a significant increase in sleep onset latency following a lower power in circadian rhythm (Kudo et al., 2011). The same model was also reported to display alterations in sleep homeostasis, including a reduction in REM sleep over a 24‐h period, increased NREM sleep during the quiescent phase and alterations in oscillatory EEG activity (McDowell et al., 2014). Some studies in the Tfam genetic‐based (MitoPark) model study found no remarkable changes in sleep latency (Langley et al., 2021), whereas others have shown that MitoPark mice have lower amplitude and higher rate of fragmentation in circadian rhythms and behavioural arrhythmia in constant conditions with ageing that develops in parallel with the progressive degeneration of midbrain dopaminergic neurons (Fifel & Cooper, 2014). Gradual reduction of endogenous dopamine levels and lower dopamine D2 receptors in dopaminergic neurons are reported as responsible for such alterations in circadian rhythms in the MitoPark model (Branch et al., 2016).

The effect of age‐related degeneration of dopaminergic neurons on the lengthening of the circadian period in the absence of circadian amplitude alteration was shown in a study with the neurotoxic MPTP mouse model, indicating that PD‐like circadian phenotypes might not be confined to decreased rhythm amplitude and/or robustness (Tanaka et al., 2012). Changes in the expression of clock genes have been shown in an animal model of PD employing 6‐OHDA, with levels of expression of *bmal1*, *per2* and *clock* genes, which were decreased in this model, but the transcription of *rorα* was increased and acetylation of *bmal1* promoter was increased due to the reduction in NAD‐dependent deacetylase sirtuin‐1 (Wang et al., 2018). In addition, it has been reported that in 6‐OHDA lesioned rats, the loss of dopaminergic neurons in the substantia nigra results in a phase advance of circadian locomotor rhythms (Souza et al., 2018), as well as a reduction in levels of *BMAL1* transcription (Li et al., 2017). In the rotenone lesion model of PD, changes in the rhythmic expression of clock genes in the SCN, such as a phase delay in *bmal1* or a phase advance in *per1* expression (Mattam & Jagota, 2015), and lower amplitude of locomotor and body temperature rhythms and greater rhythm fragmentation (Lax et al., 2012) suggest a level of circadian rhythm dysfunction that may be of functional significance in the overall phenotype of the model. Studies in neurotoxic non‐human primate models of PD reveal alterations in sleep architecture including disturbed REM sleep and excessive daytime sleepiness (Hyacinthe et al., 2014), sleep attacks and reduced activity during the day, higher sleep fragmentation and sleep onset latency at night (Choudhury & Daadi, 2018) and reduced amplitude and power of circadian rhythms in activity (Fifel et al., 2014); however, normal circadian rhythms are reported in some non‐human primate models of PD, whereas the number and total duration of daytime activity increased in the absence of detectable Parkinson pathology (Franke et al., 2016).

An important question that may be partially answered using pre‐clinical models is the extent to which circadian rhythm changes in PD models resemble those abnormalities reported in pre‐clinical models of other neurodegenerative conditions (Nassan & Videnovic, 2022). For example, many models of other neurodegenerative conditions such as Alzheimer's disease and Huntington's disease display circadian rhythm fragmentation/decreased circadian amplitude as a prominent feature, suggesting that there might be a commonality in the mechanisms resulting in such circadian rhythm changes, such neuroinflammation (Lananna & Musiek, 2020). It may be that only through the examination of several different PD models and the identification of phenotypic commonalities between these models, in terms of sleep and circadian function, can translatable insight be derived to enhance and inform the study of sleep and circadian rhythms in clinical PD populations. Further, given the high level of co‐morbidity of PD with other neurodegenerative conditions, cross‐comparison of animal models of PD and other neurodegenerative diseases may provide further insight into circadian rhythms and sleep disturbances across the clinical spectrum of PD.

## 9 FUTURE RESEARCH AGENDA

Circadian perspectives are gradually becoming a mainstream consideration in medicine (Kramer et al., 2022). As such, the examination of chronobiological processes and the application of chronotherapeutic approaches may be of clinical utility in the management of PD (Lee et al., 2021). However, to advance this important area, a conceptually and logistically coherent approach will be required to formulate and address key questions. We propose that some of these issues are as follows:

1. To systematically describe on a multi‐modal level the nature of circadian rhythm changes that precede, and occur in, PD. Such assessments may involve longitudinal samples in well‐powered studies that embrace clinical heterogeneity rather than try to ‘control’ it away. Further, delineating the circadian impacts of PD pathology from the circadian impacts of PD medication will be key in identifying practical steps that may be taken to improve circadian function and sleep health in PD patients.

The literature on circadian rhythms and PD, like in many adjacent areas, is currently dominated by relatively small studies with inconsistent protocols between studies (e.g., Huang et al., 2021). Multi‐centred interdisciplinary work may address these concerns by enabling large‐scale work, although of course funders will play a key role in enabling this. Secondary analysis of the data sets arising from prospective studies, especially in those that have included actigraphy measures at some point, may also be of use (Sambou et al., 2021; Windred et al., 2021). However, the utility of such analyses will be constrained by the construction of the protocols for these large cohorts; for example, the UK Biobank has actigraphy from 7 days from a sub‐sample of participants, whereas bespoke studies would be designed to incorporate more extensive actigraphy data. In terms of the assessment of circadian function, the use of new circadian assessment approaches, such as single time point molecular ascertainment of the circadian phase (Wittenbrink et al., 2018), may be usefully deployed alongside more established techniques.

- Clinical studies should be systemically coordinated with studies in pre‐clinical PD animal models. Animal models have great potential to augment and complement clinical studies, but their use needs to be carefully considered in the design of studies. Such work may include the adoption of standardized protocols across collaborating sites, the use of several models to uncover commonalities that may be the most relevant to PD rather than ‘one off’ observations in single models that may not well recapitulate the clinical picture of PD, the use of both male and female mice to avoid bias and the use of mice across all life stages. Furthermore, for the use of transgenic mouse model consideration needs to be given to the background strain, as there can be large differences in circadian function in wild‐type animals between commonly used in‐bred strains (Schwartz & Zimmerman, 1990). Pre‐clinical models have significant potential to reveal the nature of the links between circadian function, sleep, neurodegeneration, neuroinflammation, motor and non‐motor PD symptoms.
- The circadian system is a highly distributed one that exerts a pervasive influence across physiological systems and as such may impact PD through a variety of systems and processes. For example, much attention is currently directed to the role of the gut microbiome in brain health and disease (Cryan & Mazmanian, 2022) and more specifically the role of the gut microbiota in PD (Dong et al., 2022). The gut flora is under strong circadian control and contributes to regulating whole‐organism circadian rhythms (Teichman et al., 2020). Future work may usefully explore these interactions for novel insight into the aetiology and symptomatology of PD. Another interesting facet that may be explored is the link between type 2 diabetes and PD because type 2 diabetes patients are at increased risk of developing PD (Xxxx et al., 2022). Circadian rhythms are recognized as significant in type 2 diabetes, as circadian processes are central to the temporal organization of metabolism (Kelly et al., 2020). Furthermore, PD and diabetic complications such as retinopathy may share overlapping pathophysiological changes in circadian rhythms, α‐synuclein aggregation, dopaminergic system, neurotrophic factors release and Wnt signalling (Zhang et al., 2022). Therefore, future investigations may usefully explore the nature of the circadian‐metabolic health‐PD axis.
- How does real‐world circadian disruption affect PD risk? It is recognized that a large proportion of the population lives under social and working conditions that result in circadian rhythm disturbance (Lunn et al., 2017). For example, approximately one fifth of workers are shift workers, which results in the dysregulation of circadian processes and a heightened risk of common chronic disorders (Boivin et al., 2022). Further, non‐shift workers may experience circadian dysfunction because of conflicts between internal biological time and social time (‘social jetlag’), which is implicated as a risk factor for poorer physical and psychological health (Caliandro et al., 2021). Determination if either shift work or social jetlag serves as risk factors for developing PD would have significant public health implications.
- Further, develop and validate actigraphic and wearable device procedures and scoring algorithms for their use specifically in PD. As most actigraphic scores are validated in healthy cohorts, it is currently unclear as to how the motor symptoms of PD may impact measures derived from those processes. As such, there should be a focussed effort on validating established actigraphic procedures and new and emerging technologies specifically within the clinical context of PD.

## 10 CONCLUSION

Circadian rhythm disturbances in PD appear to be common, may be important contributors to other sleep disorders and problems in PD such as insomnia and excessive daytime sleepiness and may be exacerbated by dopaminergic therapy for PD. Such circadian dysfunctions may be tractable through the application of circadian medicine, but to advance this important area, a coherent research agenda will be needed to develop the depth and quality of the research evidence base to the point that clinical practice can be informed and advanced for better patient outcomes.

## CONFLICTS OF INTEREST

The authors declare that there are no conflicts of interest.

## AUTHOR CONTRIBUTIONS

Beverley M. Henley and Andrew N. Coogan conceived the idea. Ziba Asadpoordezaki, Andrew N. Coogan and Beverley M. Henley researched the literature and wrote the review article.

11

### PEER REVIEW

The peer review history for this article is available at https://publons.com/publon/10.1111/ejn.15859.
